# Induction of Antifungal Tolerance Reveals Genetic and Phenotypic Changes in *Candida glabrata*

**DOI:** 10.3390/jof11040284

**Published:** 2025-04-04

**Authors:** Christy Chedraoui, Nour Fattouh, Setrida El Hachem, Maria Younes, Roy A. Khalaf

**Affiliations:** 1 Department of Biological Sciences, Lebanese American University, Byblos P.O. Box 36, Lebanon; christy.chedraoui@lau.edu.lb (C.C.); nfattouh@sgub.edu.lb (N.F.); setrida.elhachem@lau.edu (S.E.H.); maria.younes01@lau.edu (M.Y.); 2 Department of Biology, Saint George University of Beirut, Beirut 1100-2807, Lebanon

**Keywords:** *Candida glabrata*, fluconazole, caspofungin, tolerance, yeast

## Abstract

*Candida glabrata* is an opportunistic, pathogenic fungus that is increasingly isolated from hospitalized patients. The incidence of drug tolerance, heteroresistance, and resistance is on the rise due to an overuse of antifungal drugs. The aim of this study was to expose a sensitive *C. glabrata* strain to sequentially increasing concentrations of two antifungal drugs, fluconazole, an azole that targets ergosterol biosynthesis, or caspofungin, an echinocandin that targets cell wall glucan synthesis. Analysis of the drug-exposed isolates showed development of antifungal tolerance, chromosomal abnormalities, decreased adhesion, attenuated virulence, and an increase in efflux pump activity. Furthermore, whole genome sequencing of all isolates exposed to different concentrations of fluconazole or caspofungin was performed to determine mutations in key genes that could correlate with the observed phenotypes. Mutations were found in genes implicated in adhesion, such as in the *AWP*, *PWP*, and *EPA* family of genes. Isolates exposed to higher drug concentrations displayed more mutations than those at lower concentrations.

## 1. Introduction

*Candida* is an asexual species of yeast notorious for causing opportunistic infections in humans [1]. In populations with healthy immune systems, the commensal yeast is part of the normal human microbiota and is kept in balance by other microorganisms from causing harm in the body [2,3]. Disruptions in the microbiota can potentially lead to candidiasis, characterized by infection and inflammation due to overgrowth of *Candida* species. The most severe infection occurs when the yeast has spread to multiple body organs through the bloodstream. This type of infection is called Invasive Candidiasis (IC) and poses a significant threat to patients in intensive care units due to their weakened immune systems. In the developed world, IC is the most common fungal disease among hospitalized patients [4]. The exact number of deaths caused by *Candida* species is difficult to approximate because critical patients diagnosed with IC infections are also ill with other co-morbidities. However, a rough estimate of the death rate is often placed around 30–60%, depending on the region [5,6].

There are at least thirty *Candida* species known to cause infections in humans [7], 95% of which are attributed to only six of the most common species: *C. albicans*, *C. glabrata*, *C. tropicalis*, *C. parapsilosis*, *C. krusei*, and *C. auris.* Historically, *C. albicans*, a yeast that has been extensively studied in our laboratory, has been the main culprit of IC. In recent years, other non-*albicans* species of *Candida*, such as *C. glabrata*, are increasingly being isolated from IC patients [8]. In some instances, *C. glabrata* as well as other non-*albicans Candida* are wrongly identified as *C. albicans* in hospital laboratories [9], so *C. glabrata* infections could be more prevalent than expected.

*C. glabrata* is a haploid yeast harboring 13 chromosomes (chromosomes A to M) [10]. *C. glabrata* is distinctive from other *Candida* species because of its inability to grow hyphal structures, which are long, branching, thread-like structures that assist *Candida* in penetrating surfaces, forming biofilms, and absorbing nutrients. *C. glabrata* only exists as blastoconidia, whether it is commensal or pathogenic [11,12].

*Candida* infections are often treated through the use of antifungal agents. The number of antifungal drug classes is limited; fungi are of the same biological kingdom as humans and share a high degree of orthology, making it a challenge to develop drugs that target fungal cells without damaging human cells [13]. Currently, there are four classes of antifungals available; azoles, echinocandins, polyenes, and allylamines. Azoles and echinocandins are mostly used to treat *C. glabrata* infections. *C. glabrata* commonly experience multi-azole resistance but an increase in frequency of echinocandin resistance has been documented. In the United States, at least 10% of *C. glabrata* isolated from the bloodstream of patients were resistant towards echinocandins; however, these rates are much lower in Asian and European countries, as well as in Australia [14].

Resistance is defined as the ability of microorganisms to thrive in the presence of an antimicrobial agent that kills them or inhibits their growth; therefore, resistance can lead to treatment failure. Resistant microorganisms grow and proliferate at antifungal concentrations above the minimum inhibitory concentration (MIC). In the context of *Candida*, resistance can occur through mechanisms such as mutations in drug target genes like *ERG11*, *FKS1*, and *FKS2*, overexpression of efflux pumps, increase in plasma membrane ergosterol content, and biofilm formation [15]. Treatment failure in *Candida* infections is not only due to the development of resistance but also involves the emergence of heteroresistance and antifungal tolerance. Heteroresistance consists of a subpopulation of yeast cells growing at antifungal concentrations above MIC while the remaining cells are drug-susceptible. Tolerance consists of yeast cells surviving antifungal exposure for a longer time without an increase in MIC. Both heteroresistance and tolerance are phenomena that can positively select for resistance [16].

Our aim was to induce resistance to the azole fluconazole and the echinocandin caspofungin by exposing the reference strain *C. glabrata* CBS138/ATCC 2001 to increasing concentrations of these antifungal agents.

Azoles are synthetic drugs that interfere with the cell membrane by targeting the production of a major sterol known as ergosterol [17,18]. Compared to azoles, echinocandins are a more novel class of antifungal agents that inhibit the synthesis of β-1,3-D-glucan, a key component of the fungal cell wall [18,19]. Azole and echinocandin tolerance are complex phenomena involving genetic and cellular changes within the fungal cell. To date, development of tolerance in *C. glabrata* is not extensively evaluated. In *Candida* spp., the genetic changes associated with tolerance are primarily chromosomal abnormalities encompassing copy number variations, aneuploidies, and loss of heterozygosity. *C. glabrata* do not experience loss of heterozygosity since they are haploid organisms. An example of chromosomal abnormality leading to antifungal tolerance in *C. albicans* is trisomy of chromosome 2 conferring caspofungin tolerance [20,21]. Moreover, several genes could be involved in antifungal tolerance development [22], and being subjected to mutations could change their tolerance profile. Cellular changes associated with tolerance mainly involve activation of stress response pathways. The major *Candida* spp. stress response pathways are the calcineurin-mediated cell response, protein kinase C cell integrity, and heat shock protein 90 (Hsp90)-mediated stress response pathways. In general, these pathways maintain cellular homeostasis, particularly cell wall integrity [23].

Antifungal resistance and tolerance are two different phenomena, but in some instances, they share some characteristics such as overexpression of drug efflux transporter genes [21]. In antifungal-tolerant *Candida* spp., the latter genes could experience an upregulation [24]. Moreover, an increase in biofilm formation can act as a barrier to antifungal entry and lead to tolerance or resistance [25].

Our initial aim was to induce *C. glabrata* resistance by exposing the reference strain *C. glabrata* CBS138/ATCC 2001 to gradually increasing concentrations of an azole, fluconazole, or an echinocandin, caspofungin, in an attempt to understand the sequential evolution of resistance. However, we induced a phenomenon resembling antifungal tolerance instead of resistance. All antifungal-exposed strains were whole genome sequenced and analyzed for changes in their genomes. In particular, we screened for mutations in key genes of known function affected by antifungal exposure or related to pathogenicity. These genes can be grouped under six categories: (1) Genes coding for the ATP-binding cassette transporters *CDR1*, *PDH1*, and *SNQ2* and their regulator *PDR1*. Exposure to antifungals, especially azoles, could mutate those genes and/or lead to their overexpression, causing drug efflux [26,27]. (2) The main *ERG* genes involved in the biosynthesis of ergosterol and their regulators *UPC2A* and *UPC2B*. Exposure to azoles could mutate these genes and disrupt the ergosterol biosynthesis pathway. Ergosterol is the most abundant plasma membrane lipid in *Candida* spp. An increase in ergosterol content could hinder antifungal entry, particularly for azoles. *ERG11* is of particular interest since it codes for lanosterol 14α-demethylase, the target of azoles [28,29]. (3) *FKS* genes coding for all the subunits of β-1,3-glucan synthase, the target of echinocandins [26]. (4) Genes coding for the main chitin synthases *CHS3* and *CHS3B*, since an increase in the content of chitin, a crucial component of *Candida* spp. cell walls, could reduce or prevent antifungal entry, particularly for echinocandins [30]. (5) All *VPS* (vacuolar protein sorting) genes since vacuoles are involved in sequestering toxic compounds including antifungals. In particular, intact *VPS15* and *VPS34* could be indirectly involved in antifungal tolerance [31]. (6) *AWP*, *EPA*, and *PWP* genes involved in adhesion and biofilm formation as well as *RAP1*, *RIF1*, *SIR2*, *SIR3*, and *SIR4*. Adhesion is an important step in stable biofilm formation and biofilms could lead to antifungal tolerance or resistance [32,33,34]. In addition to screening for mutations in the genes belonging to the latter six categories, we searched for the presence of chromosomal abnormalities. Our genomic approach was supported by phenotypic analyses evaluating virulence capacity, adhesion and biofilm-forming potential, efflux pump activity, and plasma membrane and cell wall changes. We then attempted to link key genomic changes with observed phenotypes and proposed an additional definition of antifungal tolerance.

## 2. Materials and Methods

### 2.1. Candida glabrata Isolates

A reference strain of *C. glabrata*, CBS138/ATCC 2001 (obtained from the American Type Culture Collection, Manassas, VA, USA), was used as control and was exposed to fluconazole or caspofungin. The procedure was described in [35]. Briefly, a single colony of the susceptible strain was grown overnight in potato dextrose broth (PDB) (Conda Laboratories, Madrid, Spain) at 30 °C in a shaking incubator at 100 rpm. Cell count was adjusted to 10^6^ cells that were grown in 1 mL of Roswell Park Memorial Institute (RPMI-1640) medium (Sigma-Aldrich, St. Louis, MO, USA) with either 2 µg/mL of fluconazole (Thermo Scientific, Waltham, MA, USA) or 0.19 µg/mL of caspofungin (Sigma-Aldrich, St. Louis, MO, USA). The latter drug concentrations are equivalent to the MICs in the reference strain identified by the Etest method described in Section 2.2. On a daily basis for a period of two weeks, 10 μL of *C. glabrata* culture was transferred to 1 mL of RPMI medium supplemented with fluconazole or caspofungin. At the end of the two-week incubation period, *C. glabrata* were plated on RPMI agar supplemented with fluconazole or caspofungin and one colony was taken and resuspended in RPMI supplemented with a higher concentration of fluconazole or caspofungin. Also, a colony was taken for further evaluation. The MICs of fluconazole or caspofungin for *C. glabrata* CBS138/ATCC 2001 were used as starting concentrations (FL-MIC and CS-MIC). FL-R and CS-R are isolates incubated at concentrations of fluconazole and caspofungin equal to the MIC breakpoint of resistance according to the CLSI M60 guidelines [36]. FL-I and CS-I are isolates incubated at drug concentrations halfway between the MIC of *C. glabrata* CBS138/ATCC 2001 before drug exposure and the MIC breakpoint of resistance. FL-2R and CS-2R correspond to double the drug concentrations at MIC breakpoint of resistance while CS-4R corresponds to four times the caspofungin concentration at MIC breakpoint of resistance. The concentration of drug was serially increased to 128 µg/mL fluconazole and 2 µg/mL caspofungin. The isolates in Table 1a,b were obtained and stored in 15% glycerol (Thermo Fisher Scientific, Loughborough, UK) at −80 °C.

### 2.2. MIC Determination

To differentiate between the development of antifungal resistance, heteroresistance, and tolerance, antifungal susceptibility was determined using fluconazole Etest strips (bioMérieux, Marcy-l’Etoile, France) according to the manufacturer’s instructions. Etest strips were also used to determine the MIC of caspofungin before incubating *C. glabrata* CBS138/ATCC 2001 with it. However, the broth microdilution method was carried out according to the European Society of Clinical Microbiology and Infectious Diseases [37] to determine the MIC of caspofungin of all isolates incubated with this antifungal. The 4 μg/mL starting concentration of caspofungin was twofold serially diluted, reaching a minimum concentration of 0.125 μg/mL. As positive controls, the broth microdilution data of fluconazole-resistant *C. glabrata* hospital isolates from [38] and the fluconazole- and micafungin-resistant *C. glabrata* hospital isolate Cg28 [39] were considered.

### 2.3. DNA Extraction, Sequencing and Variant Calling

DNA was extracted from freshly grown colonies using the ZR Fungal/Bacterial DNA MiniPrep^TM^ kit (Zymo Research, Irvine, CA, USA) according to the manufacturer’s instructions, with one modification. The elution buffer was switched out for one that did not contain EDTA in order to be eligible for sequencing. The extracted DNA were sent for whole genome paired-end Illumina sequencing at 90x coverage that was performed by MicrobesNG (Birmingham, UK). Microbes NG also performed genome assembly for all isolates. MicrobeNG generated their own variant calling data file that identified a multitude of mutations that was analyzed through the Integrative Genomics Viewer version 2.16.2 desktop application, which was also used to confirm our mutation data. Sequences were deposited in the National Center for Biotechnology Information (NCBI) database (www.ncbi.nlm.nih.gov) under BioProject PRJNA1135925. From the variant calling files, single nucleotide polymorphism (SNP) screening was performed for the following genes of known function that can be grouped under the six categories mentioned in the introduction: *CDR1*, *PDH1*, *SNQ2*, *PDR1*, the main *ERGs*, *UPC2A*, *UPC2B*, all *FKSs*, *CHS3*, *CHS3B*, all *VPS* genes, all *AWPs*, all *EPAs*, all *PWPs*, *RAP1*, *RIF1*, *SIR2*, *SIR3*, and *SIR4*.

### 2.4. Comparative Chromosomal Abnormality Analyses

The Yeast Mapping Analysis Pipeline (YMAP) (http://lovelace.cs.umn.edu/Ymap/) [40] was used to perform a comparative chromosomal abnormality analyses of the CS-4R and FL-2R strains versus the reference strain *C. glabrata* CBS138/ATCC 2001.

### 2.5. Adhesion Assay

Isolates were grown overnight in 5 mL of PDB at 30 °C in a shaking incubator at 90 rpm. Each isolate was then spotted onto a plate of potato dextrose agar (PDA) (Conda Laboratories, Madrid, Spain) and incubated for two weeks at 37 °C in a non-shaking incubator. Each plate was photographed, then washed under running tap water for 30 s and photographed again to compare adhesion to the agar plates [41]. The experiment was performed in biological triplicates.

### 2.6. Quantification of Rhodamine 6G Efflux

The fluorescent dye rhodamine 6G (R6G) is a substrate of yeast efflux pumps [42]; a protocol to quantify the R6G efflux to infer the expression of efflux pumps was described in [39]. First, 5.6 × 10^8^ cells of *C. glabrata* were harvested by centrifugation at 3000 rpm for 5 min from an overnight culture of PDB in a shaking incubator at 32 °C. The cells were suspended in 10 mL of 1X phosphate-buffered saline solution (PBS) and incubated for 2 h at 32 °C with gentle shaking. Then, 10 µL of 10 mM R6G (Surechem Products, Suffolk, UK) dissolved in sterile water was added and the cells were incubated for another 1 h. The cells were then washed with 1X PBS three times and then suspended in 10 mL of 1X PBS. The cells were put back in the incubator at 32 °C with gentle shaking. Next, 1 mL of the supernatant was taken at 15, 30, 45, and 60 min after a 2 min centrifugation step at 9000× *g*. The optical density was measured at 527 nm using a Genesys 10S UV-Vis spectrophotometer (Thermo Scientific, Waltham, MA, USA). The experiment was performed in biological triplicates.

### 2.7. Virulence Assay

All isolates, including the reference strain, were grown overnight in PDB at 30 °C in a shaking incubator at 100 rpm. Each isolate was injected into the tail vein of six 4-week old BALB/c female mice to measure virulence through systemic infections in a murine model of disseminated candidiasis. Each injection contained a total of 4 × 10^8^ cells of *C. glabrata* suspended in 0.2 mL of 1X PBS solution. The mice were monitored for 30 days and the number of moribund mice was counted daily. A negative control set with 1X PBS but lacking *Candida* cells was also performed [43]. Moribund mice were euthanized. Protocols followed the ethical standards of the Lebanese American University’s Institutional Animal Care and Use Committee. The study was conducted under approval code: LAU.ACUC.SAS.RK3.28/January/2022.

### 2.8. Adhesion to Human Epithelial Cells

The *C. glabrata* strains were tested for their ability to adhere to human epithelial cells, described in [44]. The human cell line used was MDA-MB-231 (obtained from the American Type Culture Collection, Manassas, VA, USA). The *Candida* isolates were grown overnight in PDB at 37 °C with shaking overnight. Around 100 cells of the control, FL-2R, and CS-4R strains were incubated with the human cell line in a 6-well microtiter plate for 90 min. Wells were washed 3 times with 1X PBS to remove non-adherent *Candida* cells and then overlain with molten PDA. The number of colonies was counted and compared to a control plate that was not washed. The experiment was performed in biological triplicates.

### 2.9. Ergosterol Quantification

Ergosterol is an important membrane sterol found in the fungal cell membrane. The protocol described in [45] was performed and is briefly described here. *C. glabrata* isolates were grown in 50 mL of PDB for 17 h in a shaking incubator at 35 °C and 100 rpm. To extract plasma membrane sterols, *C. glabrata* were pelleted at 2700 rpm for 5 min. The pellet was suspended in 3 mL of a 25% alcoholic potassium hydroxide solution, vortexed for 1 min, transferred to a glass tube, and incubated in an 85 °C water bath for 1 h. After incubation, the tubes were left to cool down at room temperature before adding to them 3 mL of *n*-heptane (Thermo Scientific, Waltham, MA, USA) and 1 mL of distilled water and vortexing them for 3 min. Then, the tubes were left at room temperature for 1 h for phase separation to occur. The upper *n*-heptane layer was transferred to a clean glass tube and incubated at −20 °C for 24 h. To quantify plasma membrane ergosterol, the upper layer obtained after incubation at −20 °C was subjected to a fivefold dilution in pure ethanol and optical density was measured by the Genesys 10S UV-Vis spectrophotometer (Thermo Scientific, Waltham, MA, USA) at 230 and 281.5 nm. Each isolate’s ergosterol content was quantified in biological triplicates according to the formulas in [45].

### 2.10. Chitin Quantification

Chitin is found in the cell wall of fungal cells. To extract the cell walls of *C. glabrata*, the protocol described by [46] was used with some modifications. *C. glabrata* isolates were grown overnight in 10 mL of PDB in a shaking incubator at 30 °C and 100 rpm. To extract cell walls, *C. glabrata* were pelleted at 4000 rpm for 5 min. The pellet was suspended in 1 mL of 5 mM Tris (pH 7.8) and transferred to a 2 mL Eppendorf tube containing three cold undrilled glass beads of 3 to 5 mm in diameter (Himedia Laboratories, Mumbai, India). The suspension experienced cycles of vortexing for 30 s alternating with an incubation for 30 s on ice for a period of 1 h. The suspension was then transferred to clean pre-weighted 2 mL Eppendorf tubes. The glass beads were washed with 0.8 mL cold 1 M NaCl (Fisher Scientific, Leicestershire, UK) the suspension was transferred to the same pre-weighed tube, and the 1.8 mL cell suspension was centrifuged at 3000 rpm for 10 min. The tube containing the pellet was weighed and the pellet weight was calculated by subtracting the weight of the empty pre-weighed tube. Cell wall proteins were eliminated by suspending the cell wall in 0.5 mL protein extraction buffer [150 mM NaCl, 100 mM Na-EDTA (Sigma-Aldrich Darmstadt, Germany), 50 mM Tris (Fisher Scientific, Leicestershire, UK), 2% SDS (Sigma-Aldrich, Darmstadt, Germany), and 8 μL per 1 mL buffer of β-mercaptoethanol (Thermo Scientific, Waltham, MA, USA); pH 7.8] per 100 mg of wet weight. Two rounds of incubation at 100 °C for 10 min, cooling down at room temperature for 5 min, and centrifugation at 3000 rpm for 5 min were carried out followed by 3 washes with distilled water. After each wash, centrifugation at 3000 rpm for 5 min was performed. After the final wash step, the pellet was suspended in 1 mL of 6 N HCl (VWR Chemicals, Radnor, PA, USA), boiled at 100 °C for 10 min, pelleted at 3000 rpm for 5 min, and suspended in 1 mL distilled water in order to quantify cell wall chitin according to the protocol in [47], briefly described here. First 0.1 mL of the suspension was mixed with 0.1 mL of 1.5 N Na_2_CO_3_ dissolved in 4% acetylacetone (Sigma-Aldrich, Burghausen, Germany) and incubated at 100 °C for 20 min. Then, 0.7 mL pure ethanol and 0.1 mL of a 1.6 g *p*-dimethylaminobenzaldehyde (SCP Science, Suffolk, UK) solution were added (*p*-dimethylaminobenzaldehyde was dissolved in 30 mL concentrated HCl and 30 mL pure ethanol). After an incubation of 1 h at room temperature, optical densities at 520 nm were measured using a Genesys 10S UV Vis spectrophotometer and concentrations of cell wall chitin were determined based on a glucosamine (Merck, Darmstadt, Germany) standard curve. The experiment was performed in biological triplicates for each isolate.

### 2.11. Biofilm Quantification

To quantify biofilm production, the protocol described in [48] was adapted with slight modifications. First, 10^7^ *C. glabrata* cells/mL were suspended in 200 μL PDB and inoculated in 96-well plates which were incubated at 37 °C and 90 rpm. One day later, 100 μL of PDB were replaced with fresh PDB and plates were incubated for 48 h at 37 °C and 90 rpm. The supernatants were discarded and the biofilms underwent fixation with 200 μL pure methanol for 15 min. After discarding methanol, biofilms were exposed to 200 μL of 1% crystal violet (Himedia Laboratories, Mumbai, India) for 5 min. Three washes with distilled water were performed to get rid of excess crystal violet. To release crystal violet trapped in biofilms, 33% acetic acid (SCP Science, Baie-D’Urfé, QC, Canada) was added to the wells and optical densities were measured at 595 nm using the Multiskan FC Microplate Photometer (Thermo Fisher Scientific, Rockford, IL, USA). The experiment was performed in biological triplicates for each isolate.

### 2.12. Statistical Analysis

All graphs were generated using GraphPad Prism version 7.00, except for Figure 3a,b, which was plotted using Microsoft Excel. Statistical analysis was performed using the GraphPad Prism version 7.00 software. For the ergosterol and chitin quantification assays as well as the adhesion to human epithelial cells experiment, the Mann–Whitney test was performed to compare the data of the isolates. For the R6G assay, an ordinary two-way ANOVA was carried out. For the biofilm quantification assay, the Friedman test coupled to the Dunn’s multiple comparison test was used. For the virulence assays survival analysis, the log-rank test for trend was adopted. The data were compared to that of the control strain. Data were only considered significant if *p*-values were equal to or below 0.05.

## 3. Results

### 3.1. MICs After Drug Exposure

Etest results for *C. glabrata* isolates incubated in increasing concentrations of fluconazole and broth microdilution results for *C. glabrata* isolates incubated in increasing concentrations of caspofungin prove that *C. glabrata* developed antifungal tolerance and not resistance. MICs of all isolates are shown in Table 2a,b as well as in the Supplementary Material (Figures S1–S6).

### 3.2. Amino Acid Substitutions

For *C. glabrata* exposed to fluconazole, mutations translated into amino acid substitutions were detected in six genes (*AWP2*, *AWP3a*, *AWP8*, *AWP9*, *AWP10*, and *PWP1*) (Table 3a), while for *C. glabrata* exposed to caspofungin, only three genes were affected (*EPA15*, *EPA16*, and *EPA22*) (Table 3b). All mutations were found in genes involved in adhesion but none of them were, to date, documented to alter the adhesive potential of *C. glabrata*. To a certain extent, an increase in number of mutations is observed upon sequential increase in antifungal concentration (Table 3a,b). No mutations were observed in all the other genes mentioned in Section 2.3 of the Materials and Methods section. This was not the case for our clinical isolates from [38,39] that experienced mutations in some of these genes.

### 3.3. Comparative SNP and Chromosomal Abnormality Analyses

**Figure 1:**
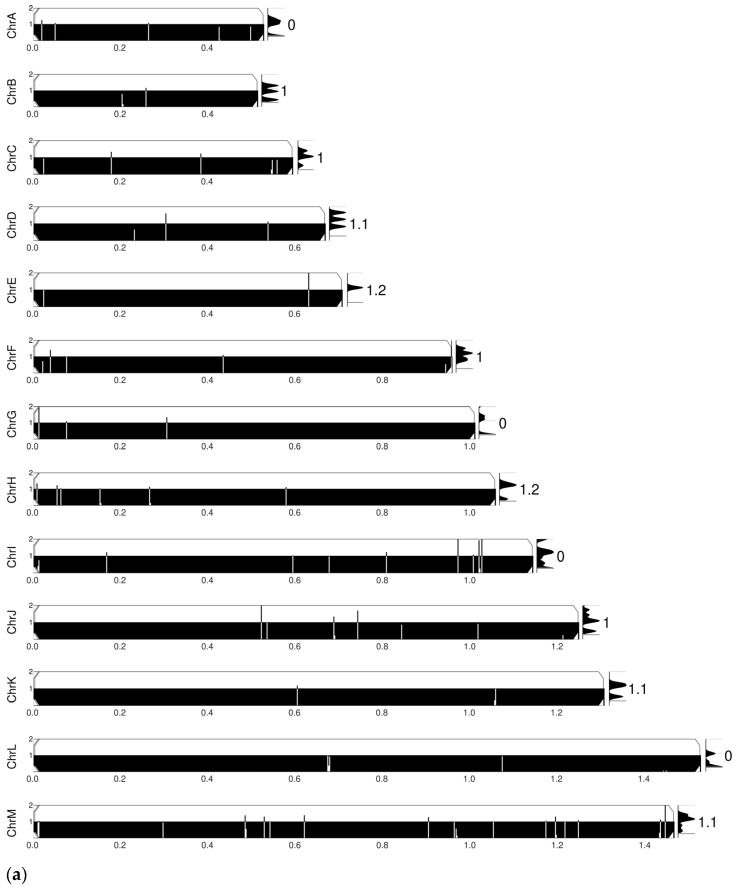

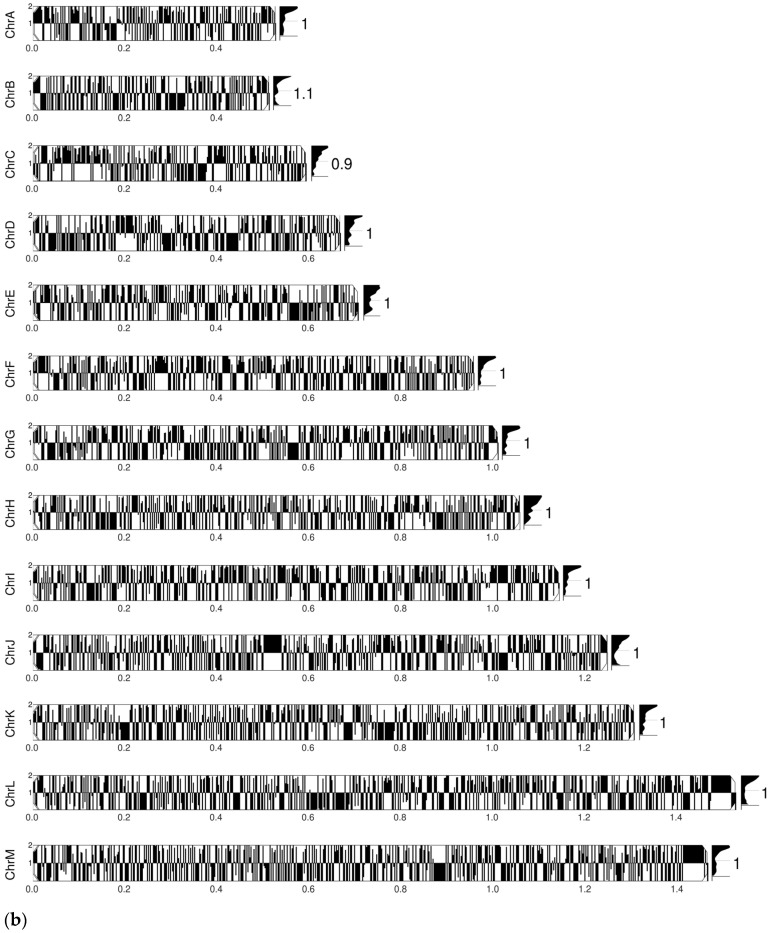
Comparison of chromosomal abnormality profiles of (**a**) FL-2R and (**b**) CS-4R. The reference genome used for comparative analyses was *C. glabrata* CBS138/ATCC 2001. Each row corresponds to a chromosome.

YMAP [40] was used for comparative chromosomal abnormality analyses of FL-2R and CS-4R versus the reference strain *C. glabrata* CBS138/ATCC 2001. In FL-2R, chromosomes A, G, I, and L experienced losses while chromosomes D, E, H, K, and M harbored amplifications. CS-4R experienced more chromosomal rearrangements compared to FL-2R, suggesting genomic instability while FL-2R exhibited genomic stability but larger scale genetic events (Figure 1a,b).

### 3.4. Decreased Adhesion in Drug-Tolerant Isolates

**Figure 2:**
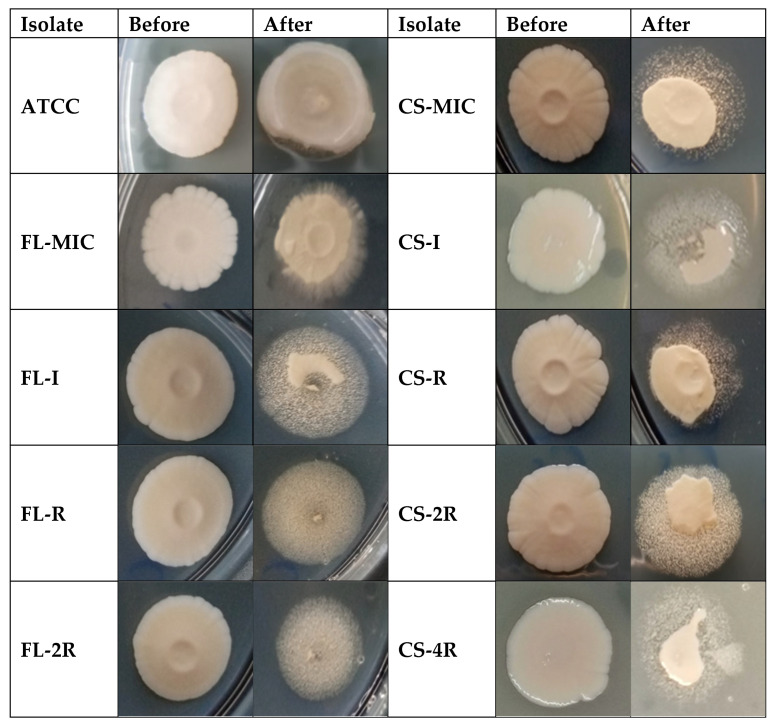
Adhesion assay. All isolates were spotted on PDA plates, left to incubate for 14 days at 37 °C, and then rinsed with running tap water. Images were taken right before washing and immediately after.

All isolates were spotted on an agar plate and photographed before and after washing with tap water. Results of the adhesion assay show a decrease in adhesion upon exposure to fluconazole or caspofungin (Figure 2).

### 3.5. Decreased Virulence in Drug-Tolerant Isolates

**Figure 3:**
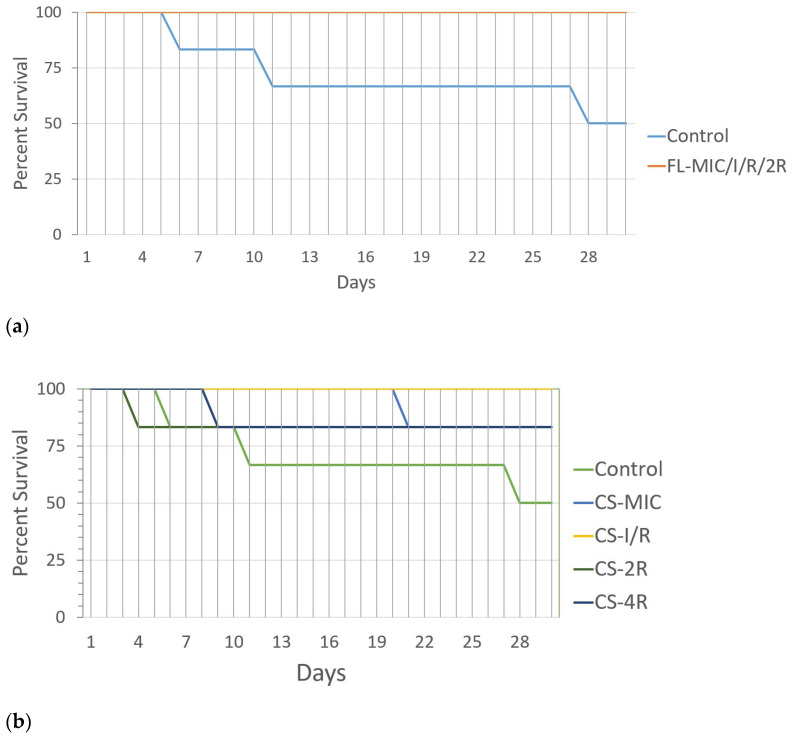
(**a**) Virulence assay for isolates exposed to fluconazole. The percent survival of mice after injection is represented as a function of days. Blue line represents the control. Each isolate was injected into a group of six mice. (**b**) Virulence assay for isolates exposed to caspofungin. Green line represents the control. Each isolate was injected into a group of six mice. Statistical significance was calculated through the log-rank test for trend: fluconazole-exposed isolates: *p* = 0.0143, caspofungin-exposed isolates: *p* = 0.2320.

Of the six mice injected with the control *C. glabrata* strain, three (50%) died in the 30-day time period. For all isolates exposed to fluconazole, no deaths were observed (Figure 3a). For all isolates exposed to caspofungin, a maximum of one mouse died in the thirty-day period (Figure 3b). The PBS 1X quality control group of mice had no deaths. *C. glabrata* is known to be less virulent compared with *C. albicans* [49]. The data suggests that the drug-tolerant isolates are less virulent than the control strain.

### 3.6. Upregulation of Efflux Pumps in Drug-Tolerant Isolates 

**Figure 4:**
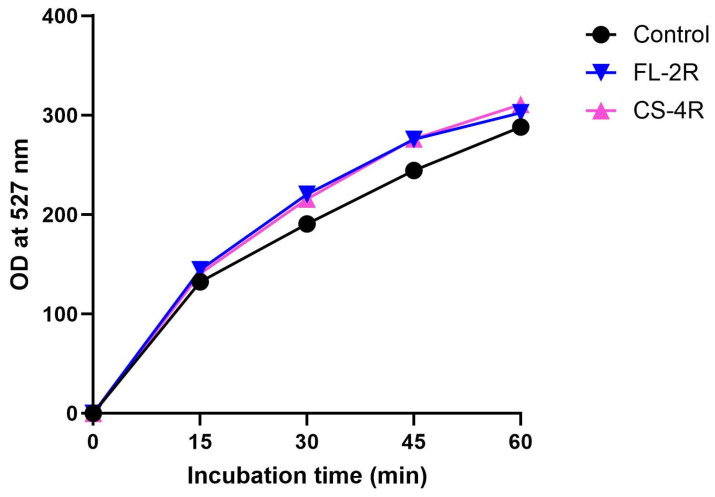
R6G assay. The black line represents the control strain. The blue line represents the isolate exposed to the highest concentration of fluconazole, FL-2R. The pink line represents the isolate exposed to the highest concentration of caspofungin, CS-4R. Statistical significance was calculated through ordinary two-way ANOVA: control vs. FL-2R: *p* < 0.0001, control vs. CS-4R: *p* < 0.0001.

Efflux pump expression was quantified and compared by the R6G assay conducted on the control isolate and the isolates exposed to the highest concentration of each drug, FL-2R and CS-4R. The drug-tolerant isolates showed a significant increase in expression of efflux pumps compared to the control isolate. At 30 min, the FL-2R and CS-4R isolates had, respectively, a 15% and 13% increase in the optical density at 527 nm compared to the control. At 45 min, both isolates had 13% increase compared to the control (Figure 4).

### 3.7. Slightly Decreased Adhesion of Drug-Tolerant Isolates to Human Cells

**Figure 5:**
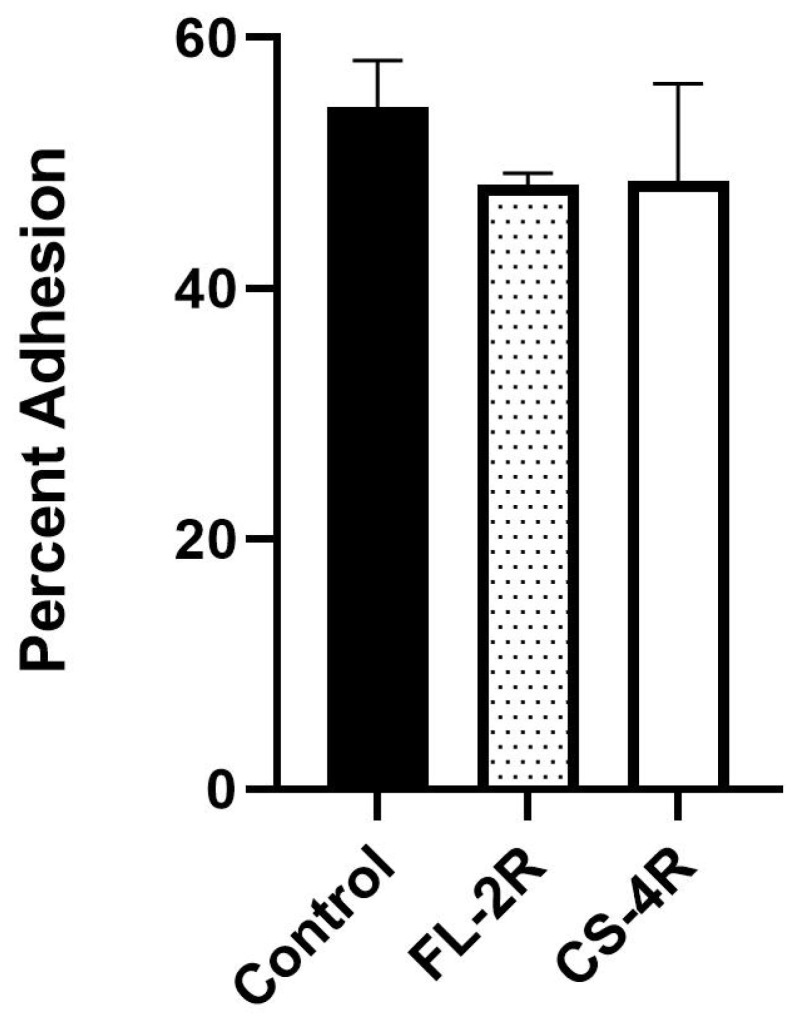
Adhesion to human epithelial cells. The black bar represents the control strain. The spotted bar represents the isolate exposed to the highest fluconazole concentration, FL-2R. The white bar represents the isolate exposed to the highest caspofungin concentration, CS-4R. A total of 54.5% of the control isolate adhered to human cells compared to 48.3% and 48.6% of FL-2R and CS-4R, respectively. Statistical significance was calculated through the Mann–Whitney test between the control and each isolate: control vs. FL-2R: *p* = 0.1, control vs. CS-4R: *p* = 0.2.

Adhesion to human epithelial cells was measured and compared for the control isolate and the isolates exposed to the highest concentrations of fluconazole and caspofungin FL-2R and CS-4R. FL-2R had a slight but consistent decrease in adhesion of around 11.5%, whereas CS-4R had a decrease of around 11% compared to the control strain, even though they did not reach the significance threshold (Figure 5).

### 3.8. No Changes in Ergosterol and Chitin Contents Upon Development of Drug Tolerance

**Figure 6:**
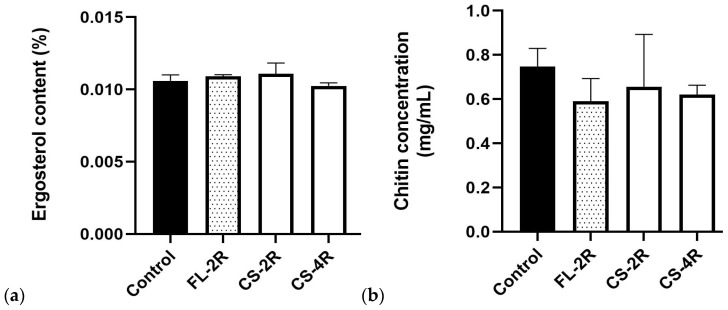
Ergosterol content and chitin content. The black bar represents the control strain. The spotted bar represents the isolate exposed to the highest concentration of fluconazole, FL-2R. The white bars represent the isolates exposed to the highest concentrations of caspofungin, CS-2R and CS-4R. (**a**) Results of the ergosterol assay; no significant changes were observed. Statistical significance was calculated through the Mann–Whitney test between the control and each isolate. Control vs. FL-2R: *p* = 0.7, control vs. CS-2R: *p* = 0.7, control vs. CS-4R: *p* = 0.4. (**b**) Results of the chitin assay represented in mg/mL of a 100 mg wet weight cell wall for the control, FL-2R, CS-2R, and CS-4R. No significant changes were observed. Statistical significance was calculated through the Mann–Whitney test between the control and each isolate. Control vs. FL-2R: *p* = 0.2, control vs. CS-2R: *p* = 0.2, control vs. CS-4R: *p* = 0.7.

The ergosterol content in the plasma membrane and the chitin content in the cell wall were quantified in the control and isolates exposed to high drug concentrations, FL-2R, CS-2R, and CS-4R. Exposure to azoles can lead to changes in the ergosterol content [50] while exposure to echinocandin does not. The ergosterol assay showed no significant changes (Figure 6a). Similarly, exposure to an echinocandin can lead to an increase in chitin content [39] while azole exposure should not have an effect. The chitin assay also showed no significant changes (Figure 6b).

### 3.9. Absence of Significant Biofilm Formation in Drug-Tolerant Isolates

**Figure 7:**
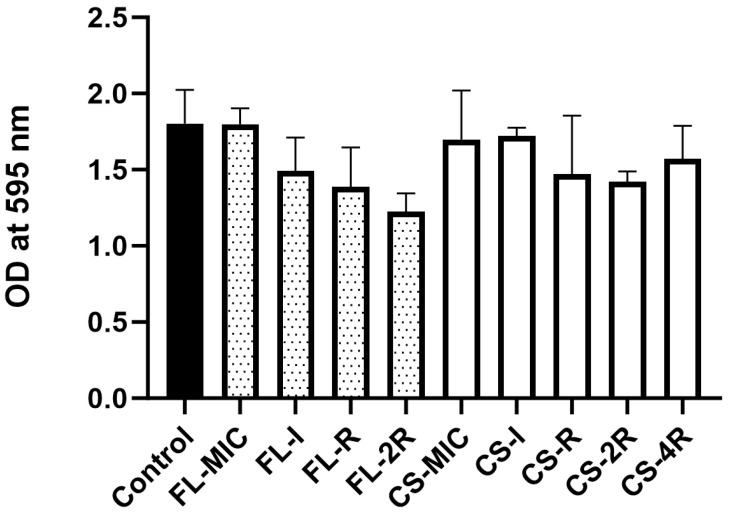
Biofilm quantification. The black bar represents the control strain. The spotted bars represent the isolates exposed to fluconazole. The white bars represent the isolates exposed to caspofungin. No significant changes were observed in the fluconazole- and caspofungin-exposed groups of isolates compared to the control. Statistical significance was calculated through the Friedman test coupled to the Dunn’s multiple comparison test. Control vs. all isolates exposed to fluconazole: *p* = 0.0664, control vs. all isolates exposed to caspofungin: *p* = 0.1339.

Biofilm quantification was performed for all isolates, and the fluconazole- and caspofungin-exposed isolates were compared to the control strain. The fluconazole- and caspofungin-exposed groups of isolates did not show any significant changes in biofilm formation capacity (Figure 7). Although not statistically significant, it is worth mentioning that fluconazole-exposed isolates have a tendency to reduce their biofilm formation potential upon exposure to gradually increasing concentrations of fluconazole. This trend is not observed in the caspofungin-exposed *C. glabrata*.

## 4. Discussion

The incidence of *C. glabrata* infection is on the rise worldwide. Unfortunately, not many studies have focused on this pathogen compared to *C. albicans*. The clinical significance of the nosocomial pathogen *C. glabrata* has recently prompted analysis of the effect of antifungal exposure on the overall fitness of this pathogen [21,49,51,52]. The wide-spread and liberal use of antimicrobials creates an enormous environment with heavy selective pressures for the development of tolerance, heteroresistance, or resistance in susceptible populations of the yeast. As exposure to antifungal selective pressures can lead to changes in the yeast’s genome and phenotype, our study attempted to expose *C. glabrata* to antifungal agents, fluconazole and caspofungin, in a laboratory environment. Exposure to drugs occurred gradually over multiple months to replicate the clinical setting. We attempted to induce antifungal resistance in the reference strain *C. glabrata* CBS138/ATCC 2001, but instead, we induced a phenomenon resembling tolerance. Tolerance is defined as survival of yeast cells for a longer time under antifungal pressure without an increase in MIC [16]. In our case, MIC slightly increased upon exposure to sequentially increasing concentrations of fluconazole and caspofungin reaching a maximum of 6 μg/mL upon exposure to 128 μg/mL of fluconazole and <0.125 μg/mL upon exposure to 2 μg/mL of caspofungin (Table 1a,b and Table 2a,b) (Figures S1–S6). *C. glabrata* were susceptible to concentrations above 128 and 2 μg/mL of fluconazole and caspofungin, respectively. MICs of 6 and <0.125 μg/mL for fluconazole and caspofungin, respectively, do not correspond to the resistance breakpoint. These results led us to propose another definition for tolerance:

Tolerance could be defined as survival of yeast cells for a longer time under increasing antifungal pressure with a slight rise in MIC without reaching the resistance breakpoint.

After tolerance was established, isolates were inspected for genetic changes. CS-4R experienced more genetic instability compared to FL-2R. These results suggest that *C. glabrata* might undergo more complex genomic changes to adapt to caspofungin compared to fluconazole. We then screened for mutations in the genes listed in Section 2.3 of the Materials and Methods section and which were described in the introduction. No mutations were detected in key genes involved in antifungal resistance, supporting the fact that we only induced tolerance in *C. glabrata*. Most importantly, mutations were not detected in the *ERG11* and *FKS* genes which are the targets of azoles and echinocandins, respectively.

Mutations were not detected in the genes coding for the efflux pumps *CDR1*, *PDH1*, and *SNQ2* or their regulator *PDR1*. However, our R6G efflux data suggest an increase in antifungal efflux (Figure 4) which could be explained by an upregulated expression of efflux pumps. Mutations were not detected in the *ERG* genes or *UPC2A* and *UPC2B,* suggesting that the ergosterol biosynthesis pathway remained intact. This is supported by the absence of changes in plasma membrane ergosterol concentrations (Figure 6a). Moreover, the absence of mutations in *CHS3* and *CHS3B* as well as the *FKS* genes imply that the cell walls remained intact, and this is partly supported by the absence of changes in cell wall chitin concentrations (Figure 6b).

Mutations in the vacuolar protein sorting *VPS* genes have been documented to be associated with antifungal tolerance and decreased virulence. In particular, intact *VPS15* and *VPS34* genes support the fact that our *C. glabrata* strains can, to a certain extent, tolerate antifungals in vivo. *VPS15* codes for a regulatory subunit of the phosphoinositide 3-kinase (PI3K) complex that activates *VPS34* coding for the catalytic subunit of the PI3K complex. Together, intact *VPS15* and *VPS34* prevent phagolysosomal maturation within macrophages [31], favoring the survival of *C. glabrata* within the host in presence of antifungal pressure. Although our antifungal-exposed strains seem to be less virulent than the reference strain in a mouse model of systemic infection (Figure 3a,b), as a follow up to this study, the burden of these *C. glabrata* infections on organs of immunocompromised mice should be assessed to better evaluate the survival endpoint.

The transcriptional silencers Rap1, Rif1, Sir2, Sir3, and Sir4, which are mainly involved in regulating the expression of *EPA1*, *EPA6*, and *EPA7*, remained intact. Usually, when these genes are disrupted, the *EPA1*, *EPA6*, and *EPA7* genes become activated and lead to an increased adhesion of *C. glabrata,* favoring the formation of stable biofilms. In our case, the decreased adhesion (Figure 2 and Figure 5) coupled with the absence of significant biofilm formation (Figure 7) are in accordance with the sequencing data. Moreover, mutations were mainly found in a few *AWP*, *EPA*, and *PWP* genes mostly associated with adherence (Table 3a,b).

Tolerance may have come about through changes in gene expression patterns, such as upregulation of efflux pumps. These pumps are essential for removing the drug from the cytoplasm of the yeast and have been reported to increase in expression in other *Candida* species. Accordingly, a R6G assay was performed to confirm this hypothesis. R6G is a highly fluorescent dye that can be easily traced to determine the transport activity of yeast membrane efflux pumps. The assay was performed on the two isolates exposed to the highest drug concentrations, FL-2R and CS-4R, and compared to the control strain. The results showed an overall increase in expression of efflux pumps in our tolerant isolates (Figure 4). Usually, upregulated efflux pumps release azoles from the intracellular to the extracellular environment, but in some cases, some efflux pumps such as Pdr1 have been reported to pump out echinocandins [53].

Upregulated ergosterol production is a documented measure against the effects of azoles in *Candida* species. This has been shown to occur in many *Candida* species; however, this adaptation mechanism is not as common in *C. glabrata* as it is in other *Candida* species, particularly *C. albicans* [50]. In fact, a recent study carried out by our laboratory did not find significant changes in plasma membrane ergosterol content in azole-resistant *C. glabrata* hospital isolates [38]. Against echinocandins, *Candida* species have been shown to engage in the process of cell wall remodeling and salvaging pathways to compensate for an impaired cell wall, such as the increase in the chitin layer of the cell wall [39]. This mechanism seems to play some role in tolerance and resistance to echinocandins in *C. albicans* but has yet to be determined as a frequent or genuine feature in *C. glabrata*. It has been established that overexpression of the chitin synthase gene can reduce the efficacy of the drug [30,54]. However, the isolates showed no mutational changes to infer these phenotypic changes. The lack of mutations in other important genes were tested for experimentally but only in the isolates exposed to the highest drug concentrations, FL-2R, CS-2R, and CS-4R, compared to the control. The results of the ergosterol quantification assay and the chitin quantification assay showed no significant increase in ergosterol content or chitin content in the drug-exposed isolates (Figure 6a,b). The isolates therefore were tolerant to fluconazole as a result of upregulated efflux pumps, not mutational changes in key genes related to the cell membrane or cell wall.

It should be noted that drug pressures faced by the isolates might be different to those faced by clinical isolates. The cells were exposed to high drug concentrations in a relatively short period of time, which is not the classical case of drug resistance in vivo that accumulates slowly over time; hence, *C. glabrata* developed tolerance to fluconazole and caspofungin. This might explain the discrepancy found in our strain mutations (Table 3a,b) versus clinical *C. glabrata* isolates resistant to azoles and echinocandins, whereby clinically resistant isolates exhibited mutations in drug resistance genes [38,39], but our isolates did not, implying tolerance in this current study.

Mutations that translated to amino acid substitutions were found in the *AWP*, *EPA*, and *PWP* family of genes in the fluconazole-exposed isolates (Table 3a). To confirm the importance of these mutations, an assay was conducted to test the isolates’ ability to adhere to plain agar. Additionally, the isolates exposed to the highest drug concentrations were tested for their ability to adhere to human epithelial cells, along with the sensitive isolate. *AWP2*, *AWP3a*, *AWP8*, *AWP9*, *AWP10*, and *PWP1* accumulated mutations in fluconazole-exposed isolates, as stated in Table 3a. The overall buildup of the mutations found in the aforementioned genes culminated in the final fluconazole-exposed isolate, FL-2R. *AWP9* demonstrated the greatest buildup of mutations, followed by *AWP10* and *PWP1* (Table 3a). The FL-2R isolate also displayed the greatest decrease in adherence to agar, as demonstrated in Figure 2. Additionally, FL-2R adhered to human epithelial cells (Figure 5) at 48.3% compared to the control’s 54.5%. The decrease in overall adherence follows a pattern of culminating mutations leading to sequential changes in the phenotypes as the exposure to fluconazole increases. In other words, the accumulation of genetic mutations led to a progressive change in the phenotype of the yeast. *AWP2* has previously been studied for its role in adhesion to polystyrene and glass; one study found that overexpressed *AWP2* led to a twofold increase in adhesiveness to polystyrene. The same study did not demonstrate the same effect for *AWP3*; the study created deletion mutants for *AWP2* and *AWP3* but only *AWP2* was found to mediate adhesion [55]. *AWP8*, *AWP9*, and *AWP10* are less characterized at the time of writing; however, one study has found that these novel adhesins are present in hyperadhesive clinical isolates [56].

For caspofungin-exposed isolates, mutations that translated to amino acid substitutions were found in *EPA1*5, *EPA1*6, and *EPA22*, as stated in (Table 3b). These genes belong to a family of cell wall adhesins, the epithelial adhesin (Epa) family of proteins. The structure of these proteins is similar to the agglutinin-like sequence (Als) proteins of *C. albicans* [57]. Up to twenty-three different genes are represented in this family of proteins. *EPA1* is known to confer adherence to mammalian surfaces while other members of the Epa family have been implicated in other virulence factors, such as biofilm formation and colonization [58]. The three mutated genes found in the caspofungin-exposed isolates are not well characterized at the time of this paper’s writing; however, their grouping into the Epa family of proteins infers their function. Similar to the fluconazole-exposed isolates, the mutations appeared to sequentially increase as the isolates were exposed to higher antifungal concentrations (Table 3b). The majority of mutations were found in the isolate exposed to the highest concentration of caspofungin, CS-4R. Adherence also sequentially decreased (Figure 2) as the isolates accumulated the mutations. CS-4R also had a noticeable decrease in adhesion to epithelial cells, having adhered to them at 48.6% (Figure 5), less than that of the control.

In general, when *C. glabrata* gains tolerance or resistance, it will suffer a fitness cost, a reduced ability to replicate and survive in a competitive environment such as in a host [49]. However, not all *Candida* pathogens follow this trend. For example, *C. parapsilosis* do not experience a decreased fitness upon development of resistance [59]. In our case, the decrease in adhesive potential (Figure 2) and the absence of changes in biofilm formation (Figure 7) compared to the control strain are proof of decreased fitness upon establishment of fluconazole or caspofungin tolerance. *C. glabrata* is considered to be less pathogenic than *C. albicans*, although it has its own arsenal of virulence factors, such as adhesins, that it may employ to establish an infection [49].

In the group of six mice injected with the control strain, there were a total of three mice dead by the end of the thirty-day period. In the isolates exposed to fluconazole, no death was recorded in the twenty-four mice injected with any of said isolates. This marks a significant drop in virulence in fluconazole-exposed isolates (Figure 3a). In the literature, gain-of-function mutations in *PDR1,* a zinc finger transcription factor, enhances virulence in *C. glabrata*. The most common mutations conferring resistance to azoles occur in *PDR1*; therefore, a positive correlation has been observed with increased resistance to azoles and virulence in murine models [60,61]. However, none of the fluconazole-exposed isolates displayed mutations in *PDR1*, nor increased virulence. This suggests that the attenuated virulence is linked to another factor. Similarly, in the caspofungin-exposed isolates, only three of the thirty injected mice died. Interestingly, deaths were recorded earlier in the time period for the isolates exposed to the highest caspofungin concentrations, CS-2R and CS-4R, occurring, respectively, at day four and day nine. The only other death to occur was at the twenty-first day for CS-MIC, suggesting an outlier. The results show attenuated virulence in the caspofungin-exposed isolates compared to the control strain (Figure 3b). Resistance to echinocandins has been shown to display decreased fitness and virulence in isolates of *Candida* species, including *C. glabrata* [61,62]. Given the marked decrease in fitness of the drug-exposed isolates, as well as confirmed mutations in adhesin genes, there appears to be a correlation between the decreased adhesion and the decreased virulence in drug-exposed isolates. Adhesion is one of *C. glabrata*’s main virulence factors; failure to attach to surfaces would mean a failure to colonize and persist in the host organism and establish disease [1,32]. As such, a decrease in adhesion (Figure 2) is mirrored by a decrease in virulence (Figure 3a,b) and no changes in biofilm formation (Figure 7). As opposed to our findings, clinical *C. glabrata* isolates exposed to selective antifungal pressure tend to be more adherent. For example, the paper published by our laboratory found that resistant isolates tend to be more adherent and showed variability in virulence potential, except for one isolate that had decreased adhesion and decreased virulence [38]. Moreover, a microevolutionary study in patients showed an increase in the adhesive properties of *C. glabrata* exposed to antifungals [63].

A similar study was recently published by our laboratory on a different *Candida* species, *C. albicans* [35]. In *C. albicans*, more mutations were detected and mutations in key genes involved in ergosterol and chitin biosynthesis such as *ERG11* and *CHS3* were observed. The effect of these mutations was confirmed by phenotypic changes in ergosterol and chitin content. *C. albicans* is a very well characterized microorganism, and the effects of resistance are well documented. Increased ergosterol production and increased chitin content are established modes of resistance in azole-resistant and echinocandin-resistant strains [64,65]. *C. glabrata* is not as understood as of yet and, despite being part of the same biological genus, may not have similar modes of resistance as *C. albicans*. *C. glabrata* is actually more closely related to the non-pathogenic baker’s yeast, *Saccharomyces cerevisiae* [57]. One noticeable difference between the two yeasts is their mechanism of resistance to azoles: *C. albicans* commonly mutates the *ERG11* gene to change the azole drug target, whereas *C. glabrata* upregulates genes involved in ABC-transporters, *PDR1* and *CDR1* [66].

This is not the first study that has exposed *C. glabrata* to antifungals in vitro and assessed the evolution of their susceptibility profiles. Shields et al. exposed *C. glabrata* to echinocandin concentrations that are three times MIC and assessed mutational frequencies, particularly in *FKS* genes. It was found that caspofungin was more potent at inducing resistance and increasing mutational frequencies compared to the two other commercially available echinocandins, anidulafungin and micafungin [67]. Cavalheiro et al. adopted a transcriptomics approach to evaluate the evolution of azole resistance upon incubation of a *C. glabrata* strain for eighty days in a culture medium supplemented with fluconazole at a therapeutic dose. This strategy established multiazole resistance and increased expression of adhesins without affecting biofilm formation [68]. Ksiezopolska et al. studied genome-wide genetic alterations in *C. glabrata* upon exposure to sequentially increasing concentrations of anidulafungin and fluconazole. *C. glabrata* rapidly adapted to these drugs at a moderate fitness cost, and evidence of cross-resistance was shown [69]. Unlike previous studies, *C. glabrata* escaped resistance in our attempt at sequentially inducing fluconazole and caspofungin resistance in vitro. Two possibilities could explain this escape from resistance. First, our strategy of in vitro resistance induction is not identical to the previously published methodologies. The strategy of Ksiezopolska et al. [69] is the most similar to our methodology since *C. glabrata* were incubated in sequentially increasing concentrations of antifungals, but the starting and final drug concentrations they adopted were different than ours. We were not able to grow *C. glabrata* at fluconazole and caspofungin concentrations above 128 and 2 μg/mL, respectively. Second, those published studies exposed clinical *C. glabrata* isolates to antifungals while we worked with the *C. glabrata* reference strain CBS138/ATCC 2001. Clinical isolates often have distinct genetic backgrounds and antifungal susceptibility profiles compared to the reference strain. They are generally more predisposed to developing resistance to antifungals since they adapted to more hostile environments, particularly if they have previously been exposed to antifungals.

In conclusion, this study induced tolerance in vitro in a *C. glabrata* isolate followed by whole genome sequencing and phenotypic analysis to better understand mechanisms of tolerance and virulence in this poorly studied organism. These fluconazole-tolerant and caspofungin-tolerant isolates were analyzed for genotypic and phenotypic changes. The isolates displayed tolerance to fluconazole as a result of upregulated expression of efflux pumps that physically remove the drug from within. Although upregulation of efflux pumps is a well-documented azole-resistance mechanism, it could also be involved in development of drug tolerance [21]. Our inability to efficiently determine the mechanism involved in caspofungin tolerance is a limitation of this study. However, more extreme chromosomal abnormalities upon exposure to caspofungin compared to fluconazole could support adaptation mechanisms leading to caspofungin tolerance. Moreover, our R6G efflux data suggests that an increased efflux could be the cause, although this is not a common mechanism of caspofungin tolerance and resistance. Although no changes in cell wall chitin contents were detected, we speculate that other cell wall components experienced changes that remodeled the cell wall architecture and led to caspofungin tolerance. No changes in cell membrane ergosterol and cell wall chitin were observed. Nevertheless, mutations were found in genes associated with adhesion, which translated to reduced overall adhesion to both agar and human epithelial cells. The inability to adhere to cell surfaces led to lower rates of murine deaths in resistant isolates and the absence of stable biofilm formation, confirm a fitness cost for gain of tolerance. Although this study sheds some light on the development of antifungal tolerance in *C. glabrata*, three main limitations need to be tackled in the future. First, an RT-qPCR experiment needs to be carried out to support the R6G data suggesting an increase in the activity of efflux pumps. Second, the complete cell wall composition and architecture need to be assessed to better understand the reason behind the development of caspofungin tolerance as well as other cellular changes implicated in antifungal tolerance. Third, changes in cellular stress response pathways favoring antifungal tolerance establishment should be evaluated. Understanding the mechanisms of antifungal tolerance is crucial for preventing its establishment, as antifungal tolerance could positively select for the development of antifungal resistance [70].

## Figures and Tables

**Table 1:** (**a**) *C. glabrata* CBS138/ATCC 2001 incubated in sequentially increasing fluconazole concentrations. (**b**) *C. glabrata* CBS138/ATCC 2001 incubated in sequentially increasing caspofungin concentrations.

**(a)**	
**Isolate Name**	**Concentration of Fluconazole (µg/mL)**
FL-MIC	2
FL-I	33
FL-R	64
FL-2R	128
**(b)**	
**Isolate Name**	**Concentration of Caspofungin (µg/mL)**
CS-MIC	0.19
CS-I	0.345
CS-R	0.5
CS-2R	1
CS-4R	2

**Table 2:** (**a**) MICs obtained following incubation at the different concentrations of fluconazole shown in Table 1a. (**b**) MICs obtained following incubation at the different concentrations of caspofungin shown in Table 1b.

**(a)**	
	**MIC (μg/mL)**
FL-MIC	4
FL-I	1.5
FL-R	7
FL-2R	6
**(b)**	
	**MIC (μg/mL)**
CS-MIC	<0.125
CS-I	<0.125
CS-R	<0.125
CS-2R	<0.125
CS-4R	<0.125

**Table 3:** (**a**) List of mutated genes detected in fluconazole-exposed isolates. The amino acid substitutions for each gene product are listed under the isolate it was found to occur in. “x” denotes that no mutation was found in that isolate. (**b**) List of mutated genes detected in caspofungin-exposed isolates. The amino acid substitutions for each gene product are listed under the isolate it was found to occur in. “x” denotes that no mutation was found in that isolate.

**(a)**						
		**Amino Acid Substitution**				
**Function**	**Gene**	**FL-MIC**	**FL-I**	**FL-R**	**FL-2R**	
**Adhesion**	*AWP2*	TT633II	TT633II	TT633II	TT633II	
	*AWP3a*	x	V64A	V64A	V64A	
	*AWP8*	x	x	N654K	N654K	
	*AWP9*	Y31D	Y31D	Y31D	Y31D	
		x	x	C312G	C312G	
		x	x	X	C267G	
		x	x	X	C132G	
		C124G	C124G	C124G	C124G	
	*AWP10*	x	x	X	C2069S	
		x	x	X	C3127S	
	*PWP1*	S6604T	S6604T	S6604T	S6604T	
		N4199K	N4199K	N4199K	N4199K	
**(b)**						
		**Amino Acid Substitution**				
**Function**	**Gene**	**CS-MIC**	**CS-I**	**CS-R**	**CS-2R**	**CS-4R**
**Adhesion**	*EPA16*	x	x	R1356G	R1356G	R1356G
	*EPA15*	x	x	x	X	P137Q
	*EPA22*	T1460P	T1460P	T1460P	T1460P	T1460P

## Data Availability

The genomic sequences generated in this study were deposited in the National Center for Biotechnology Information (NCBI) database under BioProject PRJNA1135925 at https://www.ncbi.nlm.nih.gov/bioproject/PRJNA1135925.

## Supplementary Materials

The following supporting information can be downloaded at: https://www.mdpi.com/article/10.3390/jof11040284/s1, Figure S1: Etest result of the *C. glabrata* CBS138/ATCC 2001 reference strain before exposure to fluconazole. Figure S2: Etest result of the FL-MIC isolate. Figure S3: Etest result of the FL-I isolate. Figure S4: Etest result of the FL-R isolate. Figure S5: Etest result of the FL-2R isolate. Figure S6: Etest result of the *C. glabrata* CBS138/ATCC 2001 reference strain before exposure to caspofungin.
