# Supplementary figures and images for: Induction of Antifungal Tolerance Reveals Genetic and Phenotypic Changes in Candida glabrata
Source: J Fungi (Basel). 2025 Apr 4;11(4):284. doi: 10.3390/jof11040284 (PMC12028409; doi:10.3390/jof11040284)

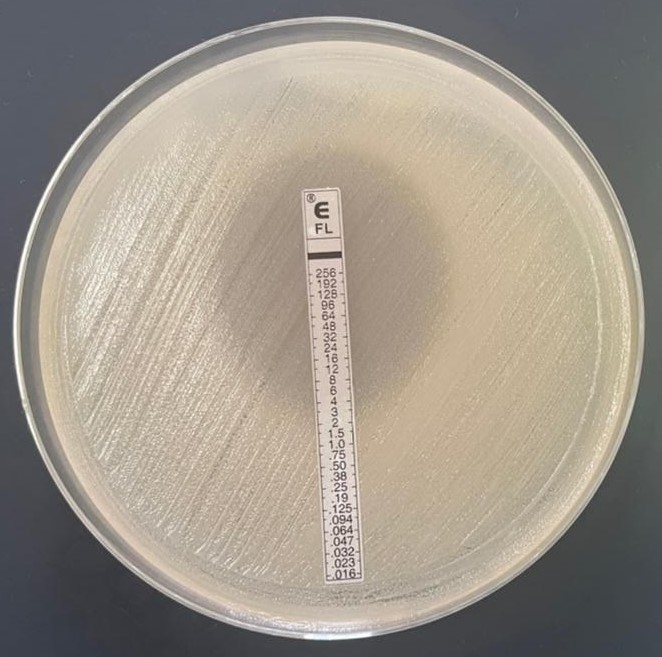

Supplement: Supplementary file 1 [file jof-11-00284-s001.zip › supplementary figures/Figure S1.jpg]

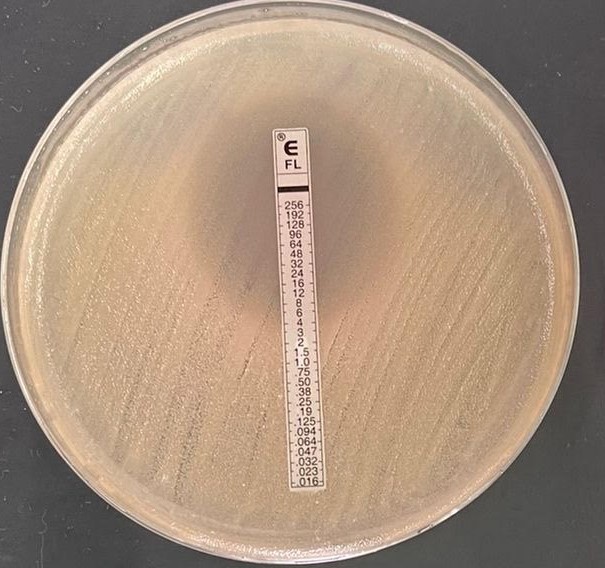

Supplement: Supplementary file 1 [file jof-11-00284-s001.zip › supplementary figures/Figure S2.jpg]

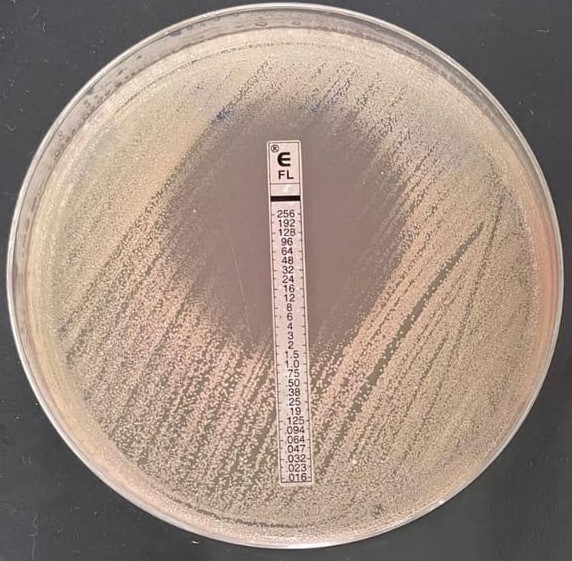

Supplement: Supplementary file 1 [file jof-11-00284-s001.zip › supplementary figures/Figure S3.jpg]

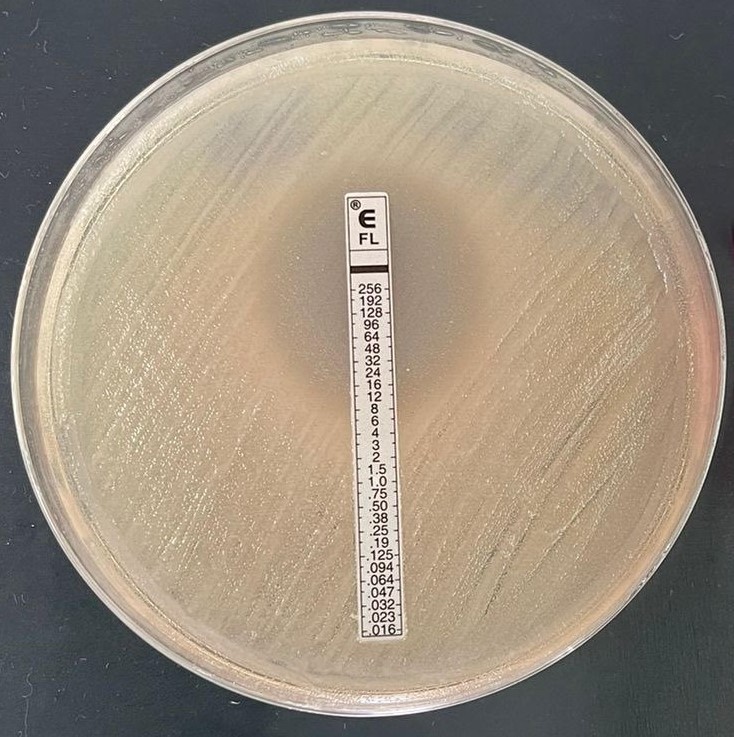

Supplement: Supplementary file 1 [file jof-11-00284-s001.zip › supplementary figures/Figure S4.jpg]

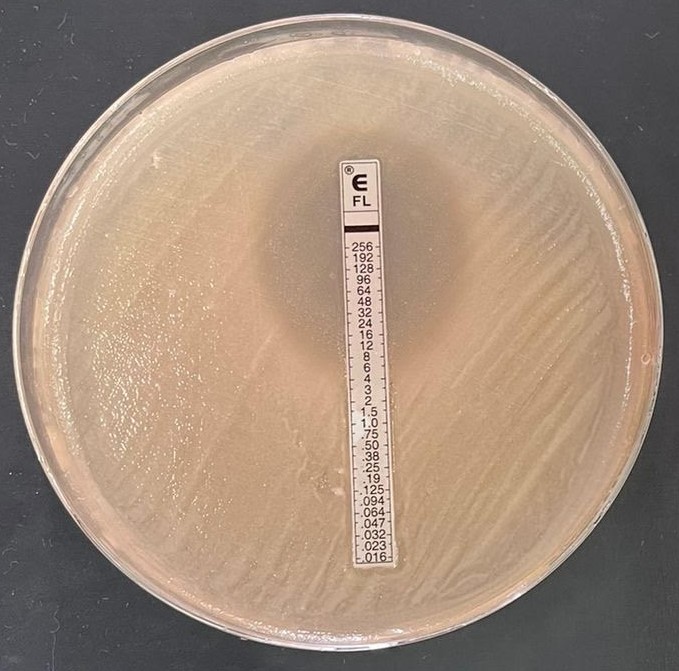

Supplement: Supplementary file 1 [file jof-11-00284-s001.zip › supplementary figures/Figure S5.jpg]

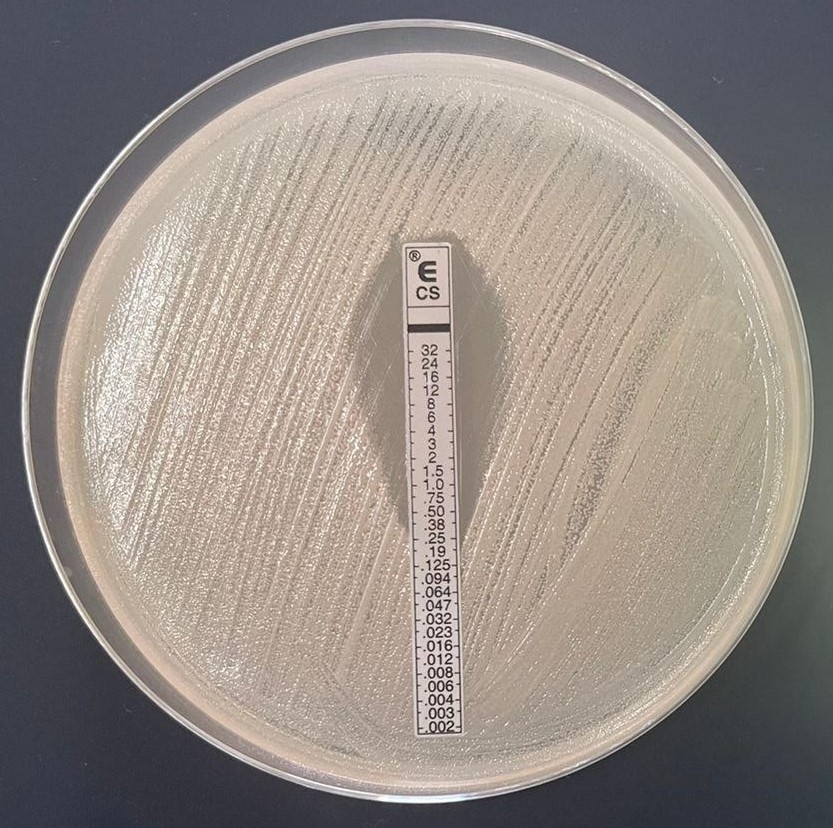

Supplement: Supplementary file 1 [file jof-11-00284-s001.zip › supplementary figures/Figure S6.jpg]
